# Influence of coronary computed tomography-angiography on patient management

**DOI:** 10.3325/cmj.2012.53.4

**Published:** 2012-02

**Authors:** Mladen Jukić, Ladislav Pavić, Jasna Čerkez Habek, Petar Medaković, Diana Delić Brkljačić, Boris Brkljačić

**Affiliations:** 1 Department of Cardiology, Sunce Clinic, Zagreb, Croatia; 2 Department of Radiology, Sunce Clinic, Zagreb, Croatia; 3 Department of Cardiology, Sestre Milosrdnice University Hospital Center, Zagreb, Croatia; 4 University of Zagreb, School of Medicine, Zagreb, Croatia; 5 Department of Diagnostic and Interventional Radiology, Dubrava University Hospital, Zagreb, Croatia

## Abstract

**Aim:**

To evaluate how coronary computed tomography-angiography (CCTA) altered the management and treatment of patients with suspected coronary artery disease (CAD).

**Methods:**

During 2009, we studied 792 consecutive patients with suspected CAD. CCTA was performed in all patients using a 64-slice dual-source CT scanner and standard scanning protocols.

**Results:**

After CCTA, obstructive CAD was excluded in 666 patients. During the 12-month clinical follow-up, 98.6% of these patients were free of major adverse cardiac events. Also, the indication for cardiac catheterization (CC) was revoked in 77.2% of patients. It was also revoked in all patients with low Morise pre-test risk, 80.7% with intermediate risk, and 72.6% with high risk. Medical therapy was changed in 54.7% of patients with confirmed CAD.

**Conclusion:**

CCTA can reliably exclude significant CAD not only in patients with low and moderate risk, but also in those with high risk. It can also reliably replace CC in the majority of elective patients regardless of risk stratification. It can also be useful in risk reclassification and optimization of medical therapy in patients with CAD.

Coronary artery disease (CAD) is the leading cause of death in most developed countries (1-3). In 50%-60% of cases, the disease is only diagnosed when the patient experiences heart attack or sudden death (2). Early detection of CAD is extremely important, so the new diagnostic methods are constantly evolving. Coronary computed tomography-angiography (CCTA) is a new, noninvasive imaging method for coronary arteries (4). It allows direct imaging of coronary artery walls and analysis of atherosclerotic plaque (5,6). Therefore, it is a useful method not only for excluding CAD, but also for the selection of optimal drug therapy (7). With current generation 64-slice scanners, CCTA can be performed in most patients with minimal patient discomfort and high diagnostic accuracy (7).

Although invasive angiography is still considered the gold standard for the diagnosis of coronary artery disease, CCTA is now recommended as a method of choice for ruling out significant CAD in patients with stable and unstable anginal syndromes with low to moderate likelihood of CAD (8). CCTA may be especially useful in patients with borderline symptoms or equivocal noninvasive testing (7).

As a transitional European country, our country until recently had a substantial lack of invasive cardiologists and equipment. Consequently, the waiting lists for cardiac catheterization (CC) were quite long. Patients on the waiting lists in whom stress-test could not be performed or with unequivocal findings on stress-test, as well as patients disinclined to undergo invasive CC, were forwarded to our institution for CCTA. The vast majority of patients were referred by cardiologists outside our institution.

In the outpatient setting, where CCTA has been used for the evaluation of patients with stable chest pain symptoms, no studies have directly measured the impact of CCTA on clinical decision-making or on patient outcomes. For these reasons, during 2009 we conducted a prospective study of clinical impact of CCTA on the management and treatment of patients with suspected CAD. The aim of this study was to evaluate if CCTA changed the management and treatment in some of these patients.

## Materials and methods

### Patient selection

The study included all patients undergoing CCTA in our institution during 2009. Signed informed consent was obtained from all patients before the investigation. Patients were eligible for the study if CAD was suspected but not previously diagnosed. A structured interview was performed before the investigation, and information about age, height, and weight of the patient, cardiac history, and current medication was collected. The following cardiac risk factors were recorded:

1. presence and degree of hypertension (for binary analysis, a systolic blood pressure >140 mm Hg was considered as abnormal regardless of antihypertensive therapy);

2. diabetes mellitus (defined as fastening blood glucose level >7 mmol/L or use of oral antidiabetic therapy or insulin);

3. smoking (defined as current smoker or previous smoker within the last year);

4. positive family history (defined as presence of CAD: myocardial infarction, coronary bypass or angioplasty, or sudden death in first-degree relatives <55 years of age if male and <65 years of age if female).

In addition, laboratory tests for total cholesterol, low-density lipoprotein, and high-density lipoprotein fractions, and triglycerides were performed. Finally, two prognostic scores were calculated: the Morise pre-test score and the Framingham risk score with the established categorical model using low-density lipoprotein cholesterol according to Wilson et al (9,10).

Both Morise and the Framingham pre-test risk scores were calculated on the basis of patients’ age, symptoms (typical angina, atypical angina, and non-anginal chest pain), medication usage, and other coronary risk factors. Risk factors include the following: current or prior cigarette smoking, history of hypertension (or antihypertensive therapy), history of insulin- or noninsulin-requiring diabetes, history of high cholesterol or taking cholesterol-lowering therapy, a family history of premature (before 60 years) coronary disease (infarction, coronary bypass or angioplasty, sudden death) in the first degree relatives, and obesity defined as a body mass index (kg/m^2^)>27. Based on Morise pre-test score, patients in the study were classified into 3 groups – with low, intermediate, and high risk (9). The study design was approved by the ethics committee of Sunce Clinic, Zagreb, Croatia.

### CT procedure

All patients were scanned on a 64-slice dual-source CT scanner (Somatom Definition, Siemens Medical Solutions, Forchheim, Germany). The detailed CT scan protocol was followed as described elsewhere (11,12). Scanning parameters were detector collimation 2 × 32 × 0.6 mm^3^, slice collimation 2 × 64 × 0.6 mm^3^ by means of a z-flying focal spot, gantry rotation time 330 ms, and pitch of 0.2-0.5 depending on the heart rate. For reduction of radiation dose exposure, an electrocardiographically gated modulation of the tube current was used in patients with stable sinus rhythm. Images were reconstructed in the mid-diastole with individually optimized position of the reconstruction window. Additional image reconstructions were performed in the end-systole if required. A data set of axial slices, multiplanar reformations, and thin-slab maximum intensity projections (5-mm thickness, 1-mm increment) was used for the analysis.

To lower the heart rate, up to 4 doses of 5 mg metoprolol were administered intravenously to patients with the heart rate ≤60 beats/min. All patients with a systolic blood pressure of at least 100 mm Hg received nitroglycerin 0.8 mg sublingually for coronary vasodilatation. Images for calcium scoring were not acquired routinely. Contrast timing was tested by an initial bolus-timing scan using 20 mL of contrast (Iopamiro 370, Bracco S.p.a, Milan, Italy), iodine content 370 mg/mL, followed by a 50 mL saline chaser. The contrast-enhanced scan was obtained using 80 to 140 mL of contrast individually adapted to the selected table feed and scan range at a rate of 4 to 5 mL/s followed by a 50 mL saline chaser. The coronary artery tree was segmented according to the modified American Heart Association classification (13). Each segment with a diameter ≥1.5 mm was evaluated visually by a single experienced reader for the degree of luminal narrowings, and rated semiquantitatively in 4 groups: <25%, 25%-49%, 50%-74%, and ≥75%. Obstructive coronary artery disease was defined as stenosis of 50% or more of the diameter of the left main coronary artery or stenosis of 75% or more of the diameter of a major epicardial or branch vessel that was more than 2.0 mm in diameter. Patients with no coronary artery disease and non-obstructive coronary artery disease were also identified.

### Findings and follow-up

The following parameters were analyzed:

1. Proportion of patients with no CAD, non-obstructive CAD, or obstructive CAD on CCTA.

2. Proportion of patients in whom an earlier indication for CC was revoked after CCTA.

3. Proportion of patients in whom CCTA led to the change in medical therapy. Optimal medical therapy for CAD (OMT) was defined according to the Clinical Outcomes Utilizing Revascularization and Aggressive Drug Evaluation (COURAGE) trial (14). OMT included antiplatelet therapy with aspirin (81-325 mg/d) or clopidogrel (75 mg/d) if aspirin intolerant. It also included lipid-lowering therapy to a target low-density lipoprotein of 2.5-3 mmol/L. Anti-ischemic therapy included beta blocator, antagonist of calcium channels, and isosorbide mononitrate, alone or in combination. Any medical therapy (AMT) for CAD was defined as incomplete or insufficiently aggressive in comparison to OMT. OMT was introduced in all patients with CAD on CCTA, where it was not previously present. Therapy upgrade was, accordingly, defined as an introduction of one or more new agents to achieve OMT. Change in the dose of already existing agent was not considered here. In patients in whom CAD was excluded on the basis of CCTA, any specific therapy for CAD was ruled out, unless it was introduced for some other reason (confirmed atherosclerosis elsewhere, hypertension, etc). Therapy reduction was, accordingly, defined as exclusion of one or more agents in patients who have been taking these medications for suspected CAD, and after CAD was not confirmed on CCTA.

4. Proportion of patients who were referred for cardiac revascularization after obstructive CAD was found on CCTA, and who received revascularization.

Twelve-month clinical follow-up period was established for two groups of patients:

a) patients with no CAD on CCTA,

b) patients with non-obstructive CAD on CCTA.

Follow-up information was obtained by direct or telephone contact. All reported events were verified by hospital records or direct contacts with the attending physician. The following clinical events were recorded in the follow-up period:

1. cardiac death;

2. nonfatal myocardial infarction;

3. unstable angina pectoris requiring hospitalization;

4. coronary revascularization not indicated on CCTA (either by bypass surgery or percutaneous coronary intervention).

All patients with obstructive CAD on CCTA were sent to revascularization. For all of them revascularization was organized within 4 weeks after the CCTA, and all had a control CC before revascularization. Due to the briefness of this period, and since their further management was organized outside of our institution, these patients were not included in the further study. Patients with obstructive CAD on CCTA, which was not confirmed at CC, were also not included in further study because their further management was performed outside of our institution.

### Statistical analysis

χ^2^ test was used to establish the relationships between pairs of categorical variables. Two-tailed test for differences between proportions was used for comparison between simple percentages. Statistical significance level was set at 0.05.

## Results

### Study population and follow-up

From January 1, 2009 to December 31, 2009, CCTA was performed in 1176 patients, 264 of whom had previously known CAD, 135 underwent a previous bypass surgery, and 129 had implanted coronary stents. Sixty-three patients were scanned due to the large blood vessels and/or valvular disease. These 327 patients were excluded from the study. In total, the study included 849 patients. In 57 patients, one or more major coronary arteries were not fully available to analysis due to the technical problems and artifacts. Further management was performed elsewhere, and these patients were not included in further analysis. There were 792 patients available for further study.

### Patients’ characteristics

Patients’ characteristics and coronary risk factor profile indicate a study population of intermediate to high risk for CAD (Table 1). The majority of patients had moderate risk (423 of 792, 53.4%), followed by those with high (270 of 792, 34.1%), and low risk (99 of 792, 12.5%). Of 513 patients previously scheduled for CC, 252 (49.1%) had high risk, 249 (48.5%) intermediate risk, and 12 (2.3%) low risk. The largest proportion of patients with AMT for CAD had high risk (246 of 270, 91.1%), followed by those with intermediate risk (369 of 423, 87.2%), and low risk (66 of 99, 66.7%).

**Table 1:** Characteristics of patients with suspected coronary artery disease

Clinical characteristics, median (range)	N = 792
Age, years	61.3 (31.4-86.5)
Male, n (%)	334 (42)
Body mass index (kg/m^2^)	27.2 (23.1-29.2)
Hypertension, n (%)	490 (63)
Smoking, n (%)	309 (39)
Diabetes mellitus, n (%)	135 (17)
Chest pain, n (%)	
typical	261 (33)
atypical	530 (67)
Positive test for ischemia, n (%)	66 (25)
Systolic blood pressure (mmHg)	145 (105-180)
Diastolic blood pressure (mmHg)	89 (60-110)
Cholesterol level (mmol/L)	6.4 (4.1-8.9)
Low density lipoprotein cholesterol (mmol/l)	4.1 (2.0-5.1)
High density lipoprotein cholesterol (mmol/L)	0.9 (0.6-1.5)
Triglycerides (mmol/L)	2.9 (0.9-7.11)
Framingham risk score (10)	13 (1-33)
Morise pre-test score (9)	13 (6-20)

### Incidence of CAD

Altogether 276 of 792 (34.8%) patients had no CAD on CCTA, 390 (49.2%) patients had non-obstructive CAD, and 126 (15.9%) patients had obstructive CAD. The majority of patients with no CAD had low risk (45 of 99, 45.5%), the majority of patients with non-obstructive CAD had intermediate risk (213 of 423, 50.3%), and the majority of patients with obstructive CAD had high risk (69 of 270, 25.5%). The correlation of CAD findings and risk stratification was significant (*P* < 0.001, χ^2^ test). However, high risk was not a sufficient predictor of obstructive CAD, because only 26% of patients with high risk actually had obstructive CAD on CCTA.

### Indication for elective CC revoked after CCTA

On CCTA, CAD was excluded in 159 of 513 patients scheduled for elective CC (30.1%), 237 (46.2%) patients had non-obstructive CAD, and 117 (22.8%) had obstructive CAD. Based on these results, indication of CC was revoked in all patients with no CAD and non-obstructive CAD, which is altogether 396 of 513 (71.9%) patients. It was also revoked in all 12 patients with low risk, in 201 of 249 (80.7%) patients with intermediate risk, and 183 of 252 (72.6%) patients with high risk. The difference between high and intermediate risk group was significant (*P* < 0.05). The significance for low risk group could not be calculated because of the small patient number.

### Changes in medical therapy after CCTA

In 495 of 792 patients (62.5%), there were no changes in medical therapy for CAD after CCTA. Therapy was modified in 297 patients (37.5%) – increased in 282 patients (35.6%) and reduced in 15 patients (1.9%).

Proportionally, therapy was increased most often in patients with high risk (156 of 270, 57.7%), followed by patients with low risk (27 of 99, 27.3%) and patients with intermediate risk (84 of 423, 19.9%). The differences between high risk group and low risk group and intermediate risk group, respectively, were significant (*P* < 0.05). The difference between low risk group and intermediate risk group was not significant (*P* > 0.05). Proportionally, therapy was most often reduced in patients with low risk (3 of 9, 33%), and not as often in patients with intermediate risk (12 out of 423, 2.8%). There were no high risk patients in whom therapy was reduced. The differences between the three risk groups in this respect were not significant (*P* > 0.05).

### Patients referred for cardiac revascularization after CCTA

Obstructive CAD was found on CCTA in 126 of 792 (15.9%) patients. All 126 patients were referred for CC. On CC, obstructive CAD was confirmed in 102 (81%) of these patients and all underwent revascularization. In 24 (19%) patients with obstructive CAD, CCTA diagnosis was not confirmed by the control CC.

### Follow up: major adverse cardiac events (MACE) after CCTA

Obstructive CAD was excluded on CCTA in 666 patients, and they were subjected to 12-month clinical follow-up. During the follow-up period, the following MACE were sought for: cardiac death, nonfatal myocardial infarction, unstable angina, and late revascularization that was not indicated on CCTA.

A total of 651 of 666 (97.7%) patients were free of MACE during the entire follow-up period (Table 2), including all 276 patients with no CAD on CCTA and 375 of 390 (96.2%) patients with non-obstructive CAD on CCTA. The difference between patients with no CAD on CCTA and patients with non-obstructive CAD on CCTA was significant (*P* < 0.001). Of patients in whom CCTA excluded obstructive CAD, all 96 patients with low risk, 360 of 369 patients with intermediate risk (97.6%), and 195 of 201 patients with high risk (97%) were free of MACE during the follow-up period (Table 2). The differences between the three risk groups in this respect were not significant (*P* = 0.26). A total of 15 patients with non-obstructive CAD on CCTA developed a MACE during the entire follow-up period; 9 had intermediate and 6 high risk (Table 3). An overall MACE rate of 2.3% during the first year after CCTA was recorded.

**Table 2:** Number of patients free of major adverse cardiac events according to the risk stratification

Morise pre-test risk (**9**)	No CAD*	Non-obstructive CAD*	Total
Low	45	51	**96**
Intermediate	156	204	**360**
High	75	120	**195**
**Total**	**276**	**375**	**651**

*Coronary artery disease.

**Table 3:** Major adverse cardiac events during the follow-up period

Major adverse cardiac events	Non-obstructive coronary artery disease
Cardiac death	0
Nonfatal myocardial infarction	0
Unstable angina	6
Late revascularization	9
**Total**	**15**

## Discussion

CCTA excluded obstructive CAD in 666 of 792 (84.1%) patients. During the 12-month clinical follow-up, 97.7% of these patients were free of MACE. All patients with no CAD and 96.2% of patients with non-obstructive CAD on CCTA were free of MACE during the entire follow-up period, and the difference between these two groups was significant (*P* < 0.05). On CCTA, CC indication was revoked in 77.2% of previously scheduled patients and medical therapy was modified in 37.5% of patients, in 94.9% of whom it was increased.

Several previous studies demonstrated a low incidence of cardiac events for patients without obstructive CAD on CCTA (15-20). Most of these studies showed an event rate below 1% per year for severe cardiac events. However, except in the study by Hadamitzky et al, all other investigations encompassed rather small or very selected populations of patients (21). Furthermore, different studies investigated various adverse cardiac events.

In our population of 666 patients with obstructive CAD excluded on CCTA, an annual event rate of MACE was 2.2%. All MACE were related to the unstable angina and late revascularization (which was not indicated by CCTA). There were no cases of nonfatal myocardial infarction or cardiac death. Among patients with no CAD on CCTA, there were no cases of MACE during the 12-month follow-up period. Significantly more MACE occurred among patients in whom CCTA revealed non-obstructive CAD. On the other hand, after CCTA excluded obstructive CAD, there was no difference in the number of MACE between the groups with different Morise pre-test risk stratification.

In a recent study on the diagnostic yield of CC on a sample of 398 978 patients, obstructive CAD was found in only 37.6% of participants (22). In our study, due to CCTA, indication for CC was revoked in 77.2% of previously scheduled patients. Moreover, indication for CC was deemed unnecessary in 72.6% of high-risk patients, and in even more patients (80.7%) with intermediate risk.

We are not aware of previous studies that examined the impact of CCTA on changes in medical therapy. In our population, 86% of patients had received some form of medical therapy for CAD before the CCTA was performed. Nevertheless, in 54.7% of patients in whom CAD was confirmed on CCTA, the therapy had to be increased to achieve OMT. The therapy was increased in significantly more patients with high risk than with low and intermediate risk.

Due to the specific situation in our country and the long waiting lists for elective CC, we included patients with somewhat higher risk than usually recommended (23) – 34.1% had high risk and only 12.5% had low risk for CAD. Nevertheless, 97.7% of patients in whom CCTA excluded obstructive CAD, and after optimization of the medical therapy, were free of MACE during the 12-month follow-up period, regardless of the pre-test risk stratification.

A limitation of the study might be that it did not include 57 patients with technically unsatisfactory CCTA, since the further management was performed elsewhere. Also, for the same reason, this study did not include patients with obstructive CAD on CCTA that was not confirmed on control CC prior to revascularization due to organizational difficulties. Although the period between CCTA and revascularization in these patients did not exceed 4 weeks, this could have affected the overall results. Also, we did not determine the occurrence of adverse cardiac events other than MACE.

Our results indicate that CCTA can consistently exclude significant CAD not only in patients with low and moderate, but also with high risk. CCTA can also replace CC in the majority of elective patients regardless of the risk stratification. In addition, they that CCTA can help reclassify risk and optimize medical therapy in patients with CAD. The potential role of CCTA in patients with high risk for CAD needs to be further explored, as well as a possible role of CCTA as a replacement for elective CC. Another area of future research may involve the analysis of how and why the findings of CCTA influence the choice of medical therapy for CAD.
